# Exhaustive Multi-Parametric Assessment of the Behavioral Array of Daily Activities of Mice Using Cluster and Factor Analysis

**DOI:** 10.3389/fnbeh.2018.00187

**Published:** 2018-08-30

**Authors:** Kenzo Yamamoto, Katsiaryna V. Gris, Jesús E. Sotelo Fonseca, Marjan Gharagozloo, Shaimaa Mahmoud, Camille Simard, Daphné Houle-Martel, Theodore Cloutier, Pavel Gris, Denis Gris

**Affiliations:** ^1^Department of Chemical Engineering and Biotechnological Engineering, University of Sherbrooke, Sherbrooke, QC, Canada; ^2^Department of Pediatrics, University of Sherbrooke, Sherbrooke, QC, Canada; ^3^Centro de Ciencias Genómicas, Universidad Nacional Autónoma de México, Cuernavaca, Mexico; ^4^Faculty of Dentistry, Department of Anesthesia, McGill University, Montreal, QC, Canada

**Keywords:** behavioral assessment, R program, supervised learning, automated behavioral assessment, homecagescan, long-term continuous recording, acclimatization, social isolation

## Abstract

Using automated supervised behavioral assessment software, we recorded and analyzed 24 h non-interrupted recordings of mice for a duration of 11 days. With the assistance of free R programming, we used correlation matrix-based hierarchical clustering and factor analysis to separate the 33 activities into meaningful clusters and groups without losing the exhaustive nature of the findings. These groups represent novel meaningful behavioral patterns exhibited by mice in home cage. Thirty-three activities were separated into 5 clusters based on dissimilarity between activities and 6 factors based on statistical modeling. Using these two methods, we describe and compare behavioral arrays of two groups of animals: 1. Continuously recorded for 11 days in social isolation and 2. Intermittently socially isolated for recording on days 1, 3, 5, 8, and 10, while socializing on the other days. This is the first work to our knowledge that interprets mouse home cage activities throughout a 24 h period and proposes a base line of a daily routine of a healthy C57Bl/6J mouse that can be used for various experimental paradigms, including disease, neuroinflammation, or drug testing to trace behavioral changes that follow intervention. In this work, we defined the necessary acclimatization period for the 24 h recording paradigm of home cage behavior. We demonstrated the behavioral changes that are associated with the effect of social isolation, intermittent socialization, and re-introduction to a familiar home cage. We provide the full description of the codes used in R.

## Introduction

The selection of behavioral tests for experimental set up presents a great challenge in behavioral neuroscience as each test taken out of context may not be informative or may even lead to the wrong conclusions. Therefore, the need for multi-parametric behavioral assessment of mice was discussed in numerous review articles (Gerlai, 2002; Kabra et al., 2013; Spruijt et al., 2014; Gris et al., 2017). Automated behavioral assessment of video recordings of mice in home cage environment provides an unbiased, unaltered reflection of mice behavior.

One of the main issues in behavioral assessment is the reproducibility of the results between laboratories (Crabbe et al., 1999; Bohannon, 2002). The oversimplified experimental design is often to blame for variation in behavioral results across laboratories (Hager et al., 2014; Spruijt et al., 2014). A detailed historical evolution of the open field testing paradigm was described by Spruijt et al. He points out that the paradigm was significantly simplified from its original form and adopted to the experiments for which it was not meant. Spruijt et al. argue that it is one of many such examples of the current behavioral assessments used in science. Moreover, long-term experiments were shown to be more reproducible compared to short-term (Fonio et al., 2012; Hager et al., 2014; Spruijt et al., 2014). This could be due to the inadequate acclimation period in short-term tests.

Automated video assessment software were used since early 2000, yet only several papers were published even from the laboratories that purchased the expensive software. Behavioral analysis software, such as Clever Sys Inc, Noldus, TSE Systems, Biobserve, etc. allow for analysis of continuous long-term (day, weeks, months) recording of home caged animals with millisecond resolution (Steele et al., 2007; Hager et al., 2014; Gris et al., 2017). It was discussed that the overwhelming amount of behavioral data that is generated by these software is difficult to handle and interpret. Until now, researchers were reporting certain activities that were chosen as pertinent to their study from these heaves of data as oppose to analyzing the full behavioral array.

Studies investigating mouse ethome are lacking, since mouse behavioral activities are usually studied one at a time using specific targeted tests (Gris et al., 2017). In our work, we investigated mouse home cage subethome using comprehensive and exhaustive behavioral analysis. Most of the commercially available behavioral software are based on supervised computer analysis, which provides exhaustive non-repetitive output of behavioral activities. It is based on a premise that a mouse is always behaving and there can be only one behavioral activity at a time. For example, if a mouse is eating, it cannot groom or if a mouse is walking, it cannot jump at the same time. Therefore, all behavioral activities are inherently linked in the output of raw behavioral data. In our manuscript, we describe one of the approaches to statistically analyze such datasets. We used R programming for multi-dimensional statistical analysis of the dataset as a necessary tool to quantify the interplay between individual behavioral activities across time and experimental conditions. Using statistical change over time in behavioral activities (grouped in clusters and factors), we defined the required acclimatization period, behavioral array changes associated with social isolation, intermittent socialization, and re-introduction to familiar home cage in C57Bl/6J male mice. The resulting multi-parametric behavioral array can serve as a baseline standard for behavioral studies of genotype differences, disease outcomes, and drug testing effects. We chose C57Bl/6J mouse as it is the most commonly used mouse in research.

## Materials and methods

### Mice

All experiments were approved by the animal ethics committee of the University of Sherbrooke. 16 C57Bl/6J mice were purchased from The Jackson Laboratory (Bar Harbor, ME). They were acclimatized for 4 weeks before the beginning of the experiments (Hoorn et al., 2011). During acclimatization, mice were housed in groups of four. Mice were maintained in a sterile environment in the animal facility with a photoperiod of 14 h of light (lights on at 6 a.m) and 10 h of dark (lights off at 8 p.m). Animals used in the experiments were 11 weeks old at day 0, housed in standard plastic cages with water and food ad libitum. For the **continuous** recording, 8 mice were placed individually into cages and kept undisturbed for the period of 11 days. For **intermittent** recording, 8 mice were placed individually into cages for recording on days 1, 3, 5, 8, 10; and were housed in groups of four during the other days. Individual cages were not changed throughout the experiment, ensuring that each mouse was re-introduced to familiar cage. Experiment was repeated twice, with 4 animals in each experimental group.

### Video recording set up

Video recording devices (Swann Pro Series HD 720P) were placed perpendicularly to the clear home cages. The cages were surrounded by three white walls to facilitate the detection of movements and to shut out any stimulus from the surroundings. Each mouse was housed individually and was visible to the camera at all times. The feeding area was located on the opposite side of the cage from the drinking spout to ensure the accuracy of detection of these activities. The cameras were connected to a Swann 8 Channel HD Digital Video Recorder. The videos were recorded in AVI format in 1-h segments and converted to MP4 format using Any Video Converter version 6.0.7.0. After the conversion, the hour-long videos were combined into 24 h segments from 13:00-13:00 using Avidemux 2.6 (32-bit). The resulting video was analyzed using the HomeCageScan (HCS) software from CleverSys Inc.

### Accuracy of the software

The settings of the software were tested by comparing HCS results with the results of three independent observers using 1 min video segments at four different time points (2 in dark cycle, 2 in light cycle). The results from the manual detection were compared with the results from the HCS software, and resulted in over 90% of concordance. Two new backgrounds for dark and light cycle were created for each 24 h segment.

### Data analysis

The HCS software analyzed the footage from the recordings. HCS is based on supervised learning (Gris et al., 2017). Data of mice' activities were expressed as the percentage of time out of 24 h and the distance traveled was expressed in meters. An original Visual Basic script was used to fix the repetition of the “Remain Low” activity that intercalated with other activities. R programming was used for factor analysis, hierarchical clustering, pie charts, parallel analysis, very simple structure (VSS), and the one- and two-way ANOVA tests to find correlations between behavioral activities and to uncover factor loadings, to cluster together activities based on their dissimilarity, to find significant differences between continuous and intermittent recording paradigms in the established factors and clusters. The *p*-value was set at 0.05 for all statistical tests.

### R and statistical significance

Correlation matrix was generated based on Pearson parametric correlation test. Hierarchical clustering matrix was computed using Euclidean distance and complete linkage method, generating a dendrogram based on dissimilarities between clusters. For the factor analysis, we used ordinary least squares technique with varimax data rotation to define the factor loadings. Statistical testing for model fit for factor analysis was conducted using the root mean square of the residuals (RMSR) ≤ 0.07 and Tucker Lewis Index of factoring reliability (TLI) ≥ 0.95 (Brunner et al., 2012). Best model was selected using VSS with RMSR ≤ 0.07, TLI ≥ 0.95, and Bayesian Information Criterion (BIC). One-way ANOVA followed by Tukey Honest Significant Difference (HSD) was used for within cluster/factor comparisons throughout 11 days period. Two-way ANOVA by Tukey HSD was used for intermittent vs. continuous comparisons for clusters/factors. The following R packages were used for analysis, statistics, and figures: *xlsx, PerformanceAnalytics, dplyr, reshape2, ggplot2, Rmisc, devtools, corrplot, nFactorspsych* (Revelle, 2017), and *GPArotation*. The full list of codes used for the data analysis using R is presented in Supplementary Material (SI 4).

## Results

### Correlation matrix

After scaling data and removing activities that were present on the list of the output results but were not detected (value of zero) during our experiments, we acquired a behavioral array of 33 activities that were exhibited by a mouse (Table 1). The results are presented in a correlation matrix as a **combined dataset**, which contains both continuous and intermittent recording results (SI 1A); **continuous dataset** only, which contains results from continuous recording paradigm (SI 1B), and **intermittent dataset** only, which contains results from the on/off recording paradigm (SI 1C). Correlation matrix represents correlations between individual behavioral activities over 11 days. We observed multiple correlations within the datasets. There are 873, 783, and 759 positive and negative correlation in combined, continuous, and intermittent datasets respectively (SI 1A–C). In the combined dataset, there are 581 positive and 292 negative correlations. In the continuous dataset, there are 555 positive and 228 negative correlations. In the intermittent dataset, there are 511 positive and 248 negative correlations. Further, the correlation between activities and time suggests that there is a strong influence of the length of isolation on behavior of a mouse. Some activities change in a similar manner to each other and, therefore, may form groups of “similar activities.” In addition, we noticed differences in patterns of correlation between combined, continues, and intermittent datasets. The results above indicate that an exploratory data analysis is necessary due to the high number of statistically significant correlations. We used hierarchical clustering to understand how individual behavioral activities relate to each other.

**Table 1:** The list of the detected activities by the software, definitions of these activities, and their corresponding abbreviations.

**Activity**	**Definition**	**Abbreviation**
Awaken	Any movement from sleep that starts and continues without resumption of sleep	awaken
Chew	Any brief period during eating where the mouth detaches from the food	chew
Come down	Any movement of the animal from a fully reared position to a position	cd
Come down from partially reared	Any movement of the animal from a partially reared up position to a low level	cd from pr
Come down to partially reared	Any movement of the animal from a fully reared up position to a partially reared up position	cd to pr
Dig	Any movement with animal's hind limbs inside bedding and resulting in a considerable movement of the bedding	dig
Drink	In a reared up position. animal's nose/mouth crosses water calibrated line	drink
Eat	In a reared up position, animal's nose/mouth crosses feeding box calibrated line	eat
Forage	Any movement with animal's forepaws and/or mouth inside bedding and resulting in a considerable movement of the bedding, typically in front of the animal	forage
Groom	Deformation of body over a defined criteria and longer than specified time	groom
Hang cuddled	Any movement of the animal resulting in animal having all four limbs at the top of the cage (more horizontal position at the top of the cage)	hc
Hang vertically from hang cuddled	Any movement from a hang cuddled position to a hang vertical position	hv from hc
Hang vertically from rear up	Any movement of the animal resulting in animal leaving the floor and not coming back down immediately and remaining vertical after leaving the floor	hv from ru
Jump	Any movement from a lower to a higher position and back to the position	jump
Land vertically	Any movement of the animal from a hanging position with feet off of the floor to the feet coming back down on to the floor	land vert
Pause	Implemented similar to sleep with those sleep constraints, but lasting only for much smaller prescribed minimum time	pause
Rear up	Any movement of the animal from a low position to a full reared up position	ru
Rear up from partially reared	Any movement of the animal from a partially reared position to a fully reared up position	ru from pr
Rear up partially	Any movement of the animal from position to a partially reared position	ru part
Remain hang cuddled	After hanging cuddled, remain in a hanging cuddled position	remain hc
Remain hang vertically	After hanging vertically remain in a vertical position	remain hv
Remain partially reared	Remain in a partially reared position	reaming pr
Remain reared up	After rearing up, remain in the reared up position	remain ru
Repetitive jumping	Any series of successive jump behaviors	repet jump
Sleep	The onset of sleep is detected at each instance there is no significant movement for a prescribed amount of time	sleep
Sniff	When the animal is either fully or partially reared, and the tip of the mouth makes some random movements (back and forth, up/down, protrude/retract)	sniff
Stationary	Any sequence for which there is no translational movement	stationary
Stretch body	Any movement from shorter to longer/elongated body (horizontally or vertically)	stretch body
Turn	Any movement of the animal from a side view to a front vice versa	turn
Twitch	Any brief movement of the animal during sleep	twitch
Walk Left	Any movement of the animal in left direction over a given distance	walk left
Walk Right	Any movement of the animal in right direction over a given distance	walk right
Walk Slowly	Any sideways movement of animal without a definite direction component	walk slowly

### Hierarchical clustering and dendrogram

**Figure 1:**
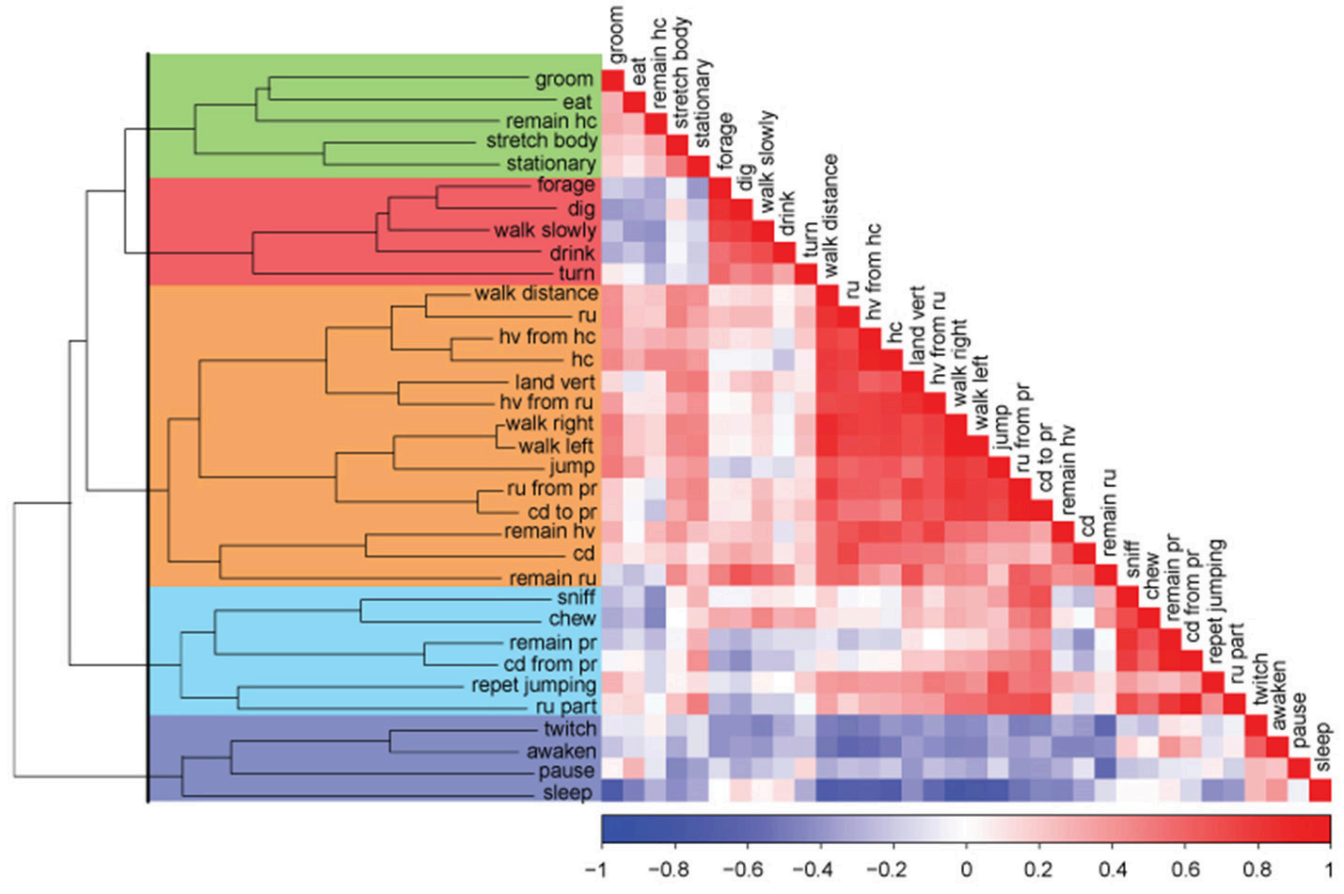
Correlation matrix-based hierarchical clustering of activities of mice recorded on both, continuous and intermittent, paradigms. Color spectrum of the graph represents positive correlations (in red) to no correlation (in white) to negative correlation (in blue) between individual activities. Dendrogram on the left groups together activities based on their dissimilarities in a bottom-up approach. The black line that cuts through the dendrogram separates the activities into five clusters (represented by colors: green, red, orange, turquoise, and blue).

Hierarchical clustering is an agglomerative approach that groups variables into clusters based on dissimilarities. To create a dendrogram, most similar clusters are grouped into new clusters of a new hierarchical order, continuing until only one cluster is left. Correlation matrix-based hierarchical clustering is one of the most widely used tools for exploratory data analysis of large datasets, such as genomic and proteomic analysis and imaging (Liu et al., 2012). For this analysis, we used the combined dataset only since it has the most variability with a mean of sum of standard deviations of 29.3 (compared to intermittent mean of sum of standard deviations of 28.5 and continuous of 19.0). For full description of descriptive statistics refer to Table 2. Hierarchical clustering matrix and the dendrogram of the combined dataset is presented in Figure 1. Hierarchical clustering matrix shows whether the correlation between behavioral activities is positive, negative, or neutral (represented by color based on Pearson correlation). Drawing of vertical line across dendrogram defines the number and the content of the resulting clusters. For example, vertical line at level 1 would separate the 33 activities in two clusters: one associated with sleep, consisting of twitch, awaken, pause, and sleep and activities that are not associated with sleep. Non-sleep associated activities can be further divided by vertical lines at levels of the lower order. The vertical line at level 4 segregates data into 5 clusters that can be explained with our current knowledge of mice behavior. We can not explain the differences in clusters beyond level four.

**Table 2:** Descriptive statistics of all activities and walking distance in continiously (Con) and intermitently (Int) recorded enimals through duration of the experiment.

	**Con**		**Int**	
**Activity**	**Mean**	***sd***	**Mean**	***sd***
Awaken	0.202079	0.050015	0.200967	0.062347
Chew	0.356458	0.148939	0.719409	0.222791
Come down	0.207571	0.0366	0.203601	0.0463
Come down from partially reared	0.967455	0.13884	1.004964	0.521486
Come down to partially reared	0.233809	0.048936	0.291991	0.120016
Dig	1.494169	0.423258	2.604185	0.834381
Drink	0.449056	0.113784	0.746845	0.209838
Eat	10.3668	1.973979	7.177402	1.25855
Forage	6.259122	1.073403	8.330143	1.524562
Groom	23.46066	1.968476	18.68524	4.26813
Hang cuddled	0.225332	0.042594	0.174274	0.062693
Hang vertically from rear up	0.091078	0.017797	0.08813	0.038712
Hang vert from hang cuddled	0.114663	0.028648	0.099533	0.035425
Jump	0.088336	0.016674	0.069721	0.044038
Land vert	0.065301	0.01588	0.071015	0.025339
Pause	3.740353	1.773996	2.86127	1.435911
Rear up	0.069514	0.01715	0.062893	0.027597
Rear up from partially reared	0.218571	0.043987	0.245906	0.092148
Rear up partially	0.51861	0.077032	0.498747	0.24107
Remain hang cuddled	6.441027	1.209611	3.759206	1.076717
Remain hang vert	0.285365	0.071791	0.262746	0.097141
Remain part reared	0.92163	0.165874	1.191883	0.810803
Remain reared up	0.536367	0.139116	0.707594	0.116328
Repet jumping	0.002267	0.001313	0.002216	0.00191
Sleep	29.20333	4.460179	36.76643	8.333053
Sniff	0.953645	0.275876	1.687088	1.000141
Stationary	0.040267	0.020495	0.029046	0.023823
Stretch body	0.627184	0.46529	0.313538	0.332001
Turn	0.859611	0.176836	0.991752	0.180759
Twitch	3.958511	0.914709	3.323925	0.737261
Walk left	0.304611	0.09113	0.2669	0.163408
Walk right	0.352656	0.090628	0.31538	0.1598
Walk slowly	0.297184	0.061412	0.581957	0.150368
Walk distance	268.5736	47.55611	248.363	78.21884

In Figure 1, the bottom cluster highlighted in blue consists of sleep, twitch, awaken, and pause activities. This cluster is the most dissimilar compared to the rest of the clusters because it is separated from the rest at the very first level of the dendrogram. We will call this cluster **sleep-related cluster** throughout this manuscript, since all activities are sleep related. The second most dissimilar group is highlighted in turquoise. It consists of sniff, remain partially reared, chew, reared up partially, repetitive jumping, and come down from partially reared activities. This cluster is separated from the rest of the data at the second level of the dendrogram. We will call this group **exploratory-like cluster** since these activities are related to exploring the environment. The next cluster contains the largest number of activities and is highlighted in orange. It consists of walking distance, reared up, hanging vertically from hanging cuddled, hanging cuddled, landing vertically, hanging vertically from reared up, walking right/left, jumping, reared up from partially reared, come down to partially reared, remain hanging vertically, come down, and remain reared up. All these activities relate to being physically active, as such, we will call this group **physically demanding cluster**. The last two clusters are the most similar according to the dendrogram. Highlighted in red are forage, dig, drink, walk slowly, and turn activities. We will call this group the **habituation-like cluster**. Highlighted in green is a cluster, which consists of stretch body, groom, remain hang cuddled, stationary, and eat activities. Since the majority of these activities are related to taking care of oneself, we will call this group the **nourishment cluster**. We will be using these clusters to assess the effect of acclimatization, social isolation, intermittent socialization, and re-introduction to familiar home cage on mice home cage behavioral array.

The variability in the data that resulted from the two experimental paradigms allowed us to explore the behavioral profile of a healthy C57BL/6J mouse and divide the 33 activities into five clusters by their association to each other.

### Factor analysis

**Figure 2:**
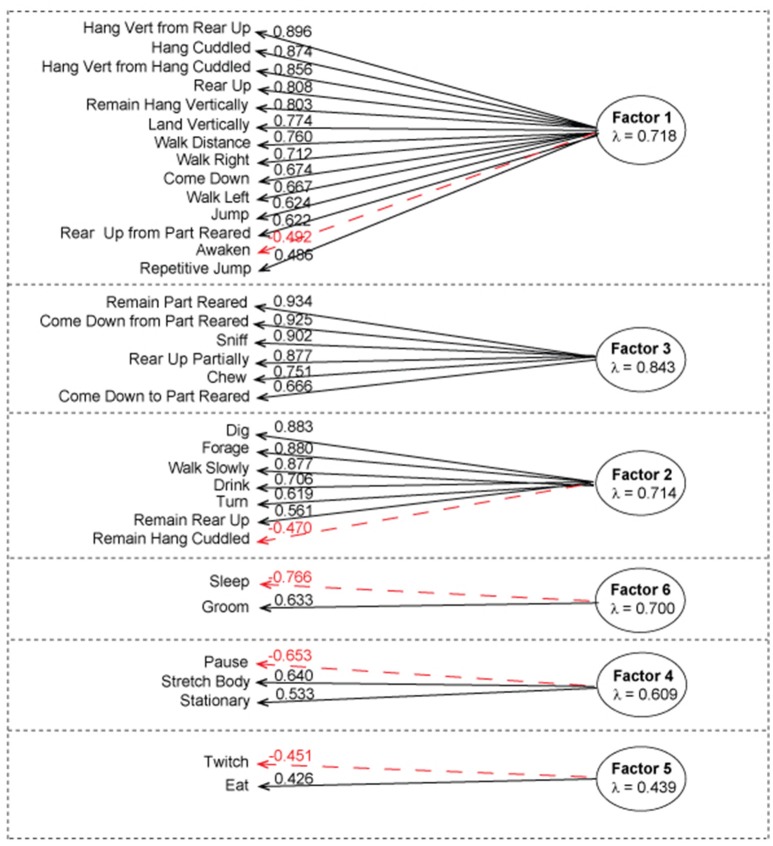
Factor analysis of the complete dataset using first order factor model. According to parallel analysis, six factors were selected for the factor analysis. The above diagram represents factor loadings on behavioral activities with the corresponding λ values. Positive correlations with the corresponding λ value loading are represented in black. Negative correlations with corresponding value loading are represented in red. Average λ of absolute values is under each factor heading. Factor one contained 14 activities that associated with physically demanding activities, factor 3 contained 6 activities associated with exploratory-like activities, factor 2 contained 7 activities associated with Habituation-like activities, factor 4 contained 3 activities associated with pre/post rest activities, factor 5 contained 2 activities associated with eating and twitching, factor 6 contained 2 activities associated with grooming and sleeping.

Factor analysis allows to estimate the unobserved structure underlying the variations of observed variables and their interrelationships (Matsunaga, 2010). We performed factor analysis on the combined dataset to generate a model, which will be used to demonstrate the relationships between activities and the variations associated with acclimatization, isolation, intermittent socialization, and re-introduction to home cage (Figure 2). While drawing of a vertical line through the dendrogram of hierarchical clustering is based on our previous knowledge of mouse behavior, factor analysis separates the data based on statistical modeling.

To establish the number of factors, we used parallel analysis, minimal residual method, which revealed that there are six factors and six components in the combined dataset (SI 2). In addition, we ran the VSS, oblmin method to verify the most appropriate number of factors based on the RMSR, TLI, and BIC, which confirmed that six factors would be the best model fit. With a loading cutoff of 0.4, we have generated six groups of activities (Figure 2). The values of the standardized value loadings range from λ = 0.934 to λ = 0.426; the mean lambda of absolute values for factor 1 is 0.718, factor 2 is 0.714, factor 3 is 0.843, factor 4 is 0.609, factor 5 is 0.439, and factor 6 is 0.700. Factor 3 is the most well-defined factor with lambda of 0.843 and factor five is the least well-defined factor with lambda of 0.439. Furthermore, there were no detected correlations between the latent constructs, which demonstrates that there is very little common variance between the defined factors. The model explains 78% of variance in the dataset. Most factors load uniquely onto one activity when a cutoff value of λ = 0.4 is used (SI 3). Furthermore, negative factor loadings represent negative correlation between an activity as it relates to other activities in a group (red colored arrows in Figure 2).

Factor 1 mainly loaded onto physically demanding activities. The activities in this group relate closely to the physically demanding cluster that was uncovered in cluster analysis. With λ = −0.492, awaken has an inverse relationship with the groups as a whole. We will call this group **Factor 1: physically demanding activities**.

Factor 2 loaded on all activities that were present in habituation-like cluster. With two additions: remain rear up activity is a part of this group and the remain hang cuddled is negatively related to the group with λ = −0.470. We will call this group of activities **Factor 2: Habituation-like activities**.

Factor 3 loaded onto activities present in the exploratory-like cluster with one substitution. Repetitive jumping present in the cluster was switched with coming down to partially reared in the factor 3 loading. The rest of the activities are common in the two groups with no negative correlations. We will call this group **Factor 3: exploratory-like activities**.

Factor 4 loaded onto pause, stretch body, and stationary activities. There is no similar cluster. Pause has an inverse relationship to the group. We observed these behaviors in mice before falling sleep (pause) and right after awaking (stretch body). Stationary behavior constitutes a very small fraction of the overall behavior of a mouse, as such, we have not observed it enough to connect it with this factor. We will call this group **Factor 4: pre/post-rest activities**.

Factor 5 is the least defined factor with an average λ of absolute values of 0.439. Twitch and eat are the only two activities in this group; with twitch inversely relating to the eating activity. We will call this group **Factor 5: eating and twitching**.

Factor 6 is a well-defined factor, which consists of grooming and sleeping activities. Sleep inversely relates to grooming. We will call this group **Factor 6: grooming and sleeping**. These two activities are predominant in mouse everyday life, constituting about 25% of 24 h period for grooming and 30% of 24 h period for sleeping.

### Video validation

If several activities are grouped together, this suggests that these activities are related. Therefore, we can reasonably expect that such activities will coincide in time. To verify this hypothesis, we watched video recordings of the experiment trying to find sequential occurrences of behavioral activities from the same group. Indeed, we found that activities in each group are often exhibited by an animal in a sequence. For example, a representative video clip 1, shows the execution of activities that are listed in the physically demanding cluster and factor 1. Video clip 2 shows the execution of exploratory-like cluster and factor 3. Video clip 3 shows habituation-like cluster or factor 2 activities being executed by a mouse. Video clip 4 demonstrates nourishment cluster activities and factor 5. Video clip 5 demonstrates activities in the sleep-related cluster and the negatively correlated activities in factors 1, 4, 5, and 6: awaken, pause, twitch, and sleep, respectively (Supplementary Videos 1–5).

### Acclimatization period

**Figure 3:**
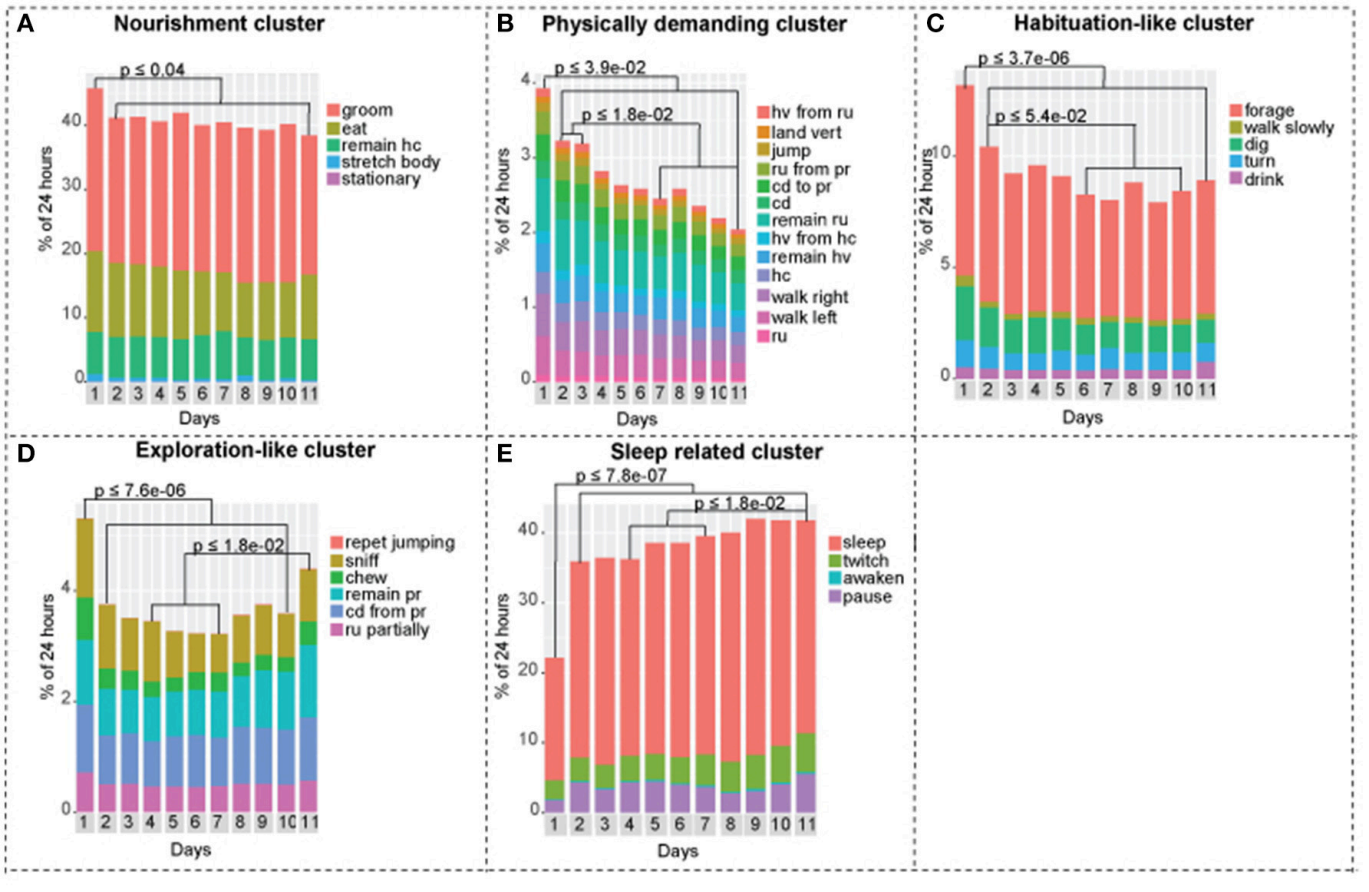
Activities of the continuously recorded mice separated into five clusters**. (A)** Nourishment cluster. Day 1 is statistically different from days 1-2-3-4-5-6-7-8-9-10-11. **(B)** Physically demanding cluster. Day 1 is statistically different from days 1-2-3-4-5-6-7-8-9-10-11, days 2-3 are statistically different from days 7-8-9-10-11. **(C)** Habituation-like cluster. Day 1 is statistically different from days 1-2-3-4-5-6-7-8-9-10-11, day 2 is statistically different from days 6-7-8-9-10. **(D)** Exploration-like cluster. Day 1 is statistically different from days 1-2-3-4-5-6-7-8-9-10, days 4-5-6-7 are statistically different from day 11. **(E)** Sleep-related cluster. Day 1 is statistically different from days 1-2-3-4-5-6-7-8-9-10-11, days 4-5-6-7 are statistically different from day 11. One-way ANOVA followed by Tukey Honest Significant Difference (HSD) test was used. *n* = 8.

**Figure 4:**
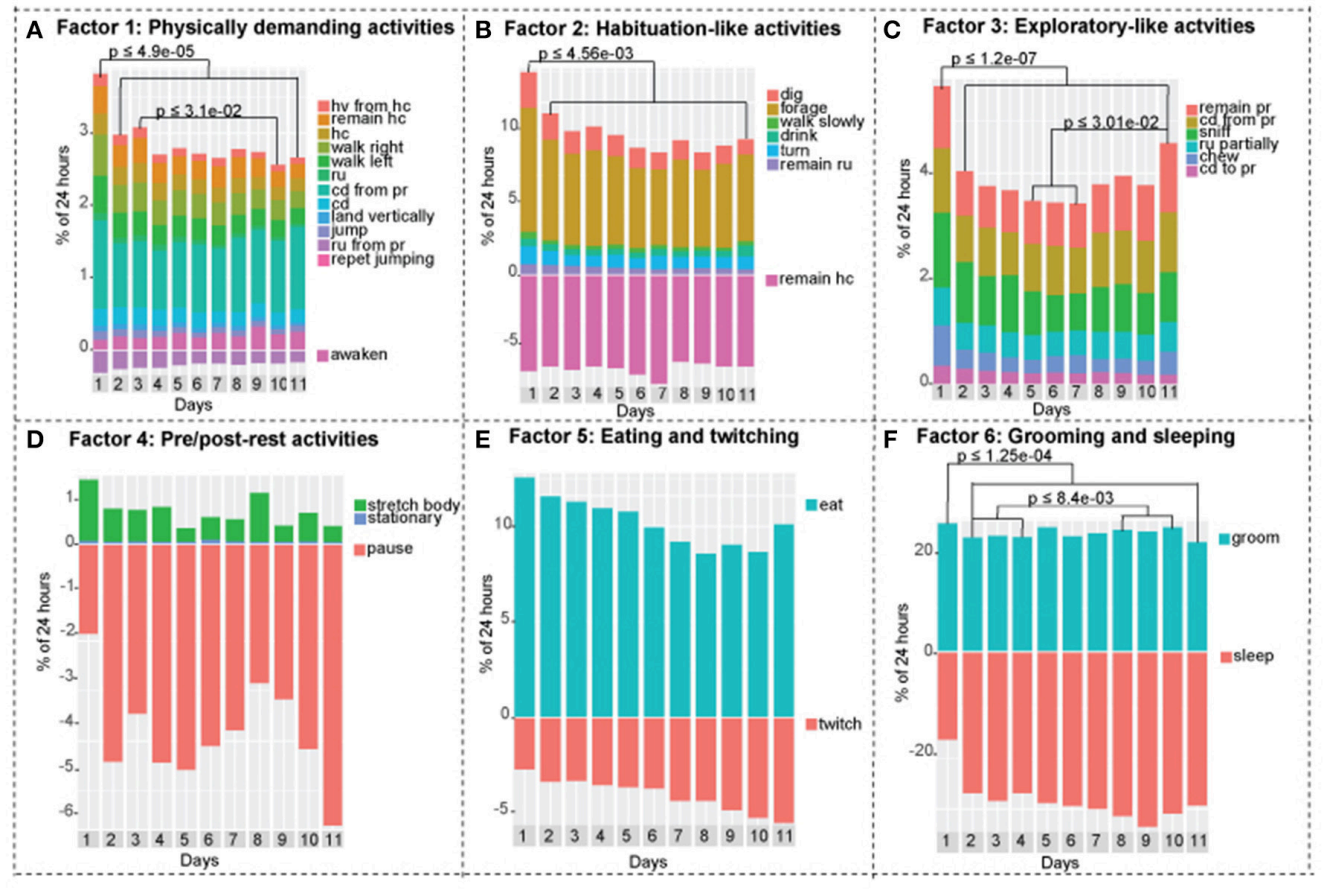
Activities of the continuously recorded mice separated into factors. Negative y-axis in factor 1, 2, 4, 5, and 6 represents inverse relationships. **(A)** Factor 1: physically demanding activities. Day 1 is statistically different from days 2-3-4-5-6-7-8-9-10-11, day 3 is statistically different from day 10. **(B)** Factor 2: habituation-like activities. Day 1 is statistically different from days 2-3-4-5-6-7-8-9-10-11. **(C)** Factor 3: exploratory-like activities. Day 1 is statistically different from days 2-3-4-5-6-7-8-9-10-11. Day 11 is statistically different from days 5-6-7. **(D)** Factor 4: pre/post-rest activities. There are no statistical differences between days. **(E)** Factor 5: eating and twitching activities. There are no statistical differences between days. **(F)** Factor 6: grooming and sleeping activities. Day 1 is statistically different from days 2-3-4-5-6-7-8-9-10-11, days 2-3-4 are statistically different from days 8-9-10. One-way ANOVA followed by Tukey Honest Significant Difference (HSD) test was used. Adjusted p value is presented on each graph accordingly. *n* = 8.

To uncover the length of time required for acclimatization, we separated the continuous dataset into clusters (Figure 3) and factor-groups (Figure 4) that were uncovered in the sections above. In both, clusters and factors, day 1 was the most dissimilar day compared to the following 10 days. In addition, we ran one-way ANOVA followed by Tukey HSD, which found significant difference between day 1 and days 2-3-4-5-6-7-8-10-11 in all clusters and in factors 1, 2, 3, and 6. As such, we conclude that on day 1 of solitary confinement in a new cage a mouse exhibits behavior that is not representative of its behavior thereafter. These patterns of behavior are representative of acclimatization. Following are the behavioral variations observed that are associated with separation from other cage mates into a new home cage environment.

Mice exhibited lesser tendency to rest, which is demonstrated by lower overall performance of activities in the sleep related cluster. On day 1, mice slept +/– 45% less compared to days 2–11. On the other hand, we observed a 1.6-fold increase in exploration-like cluster, a 1.6-fold increase in habituation-like cluster, a 1.4-fold increase in physically demanding cluster, and a 1.2-fold increase in nourishment cluster compared to the following days. All the changes are statistically significant based on one-way ANOVA followed by Tukey HSD. Similar trend is present in factors: factor 1 (physically demanding activities), factor 2 (habituation like activities), factor 3 (exploratory like activities) are all significantly increased in day 1 compared to days 2–11.

### Social isolation

Social isolation is not a black and white phenomenon. Mice do not become lonely suddenly. We observed gradual decreases and increases in mouse behavioral activities over 11 days that show trends, that reflect the effect of its new living conditions (Figure 3). We observed a gradual decrease in the physically demanding cluster (Figure 3B), a gradual decrease in habituation-like cluster (Figure 3C), a gradual decrease followed by an increase in the exploration like cluster (Figure 3D), a gradual increase in the sleep-related cluster (Figure 3E). There was no significant change in the nourishment cluster (Figure 3A). *P*-values are presented in each corresponding bar graph in Figure 3. These trends depict a timeline of the effect of isolation on daily routine of a mouse socially isolated in its home cage. Factors show similar trends. Factor 3: exploratory-like activities show an increase on day 11 (Figure 4C). Factor 6: grooming and sleeping activities increase from days 2-3-4 to 8-9-10 (Figure 4F). Factors 2, 4, and 5 had no significant change from day 2 to 11 (Figures 4B,D,E).

### The effect of intermittent socialization, re-introduction to home cage, and handling

**Figure 5:**
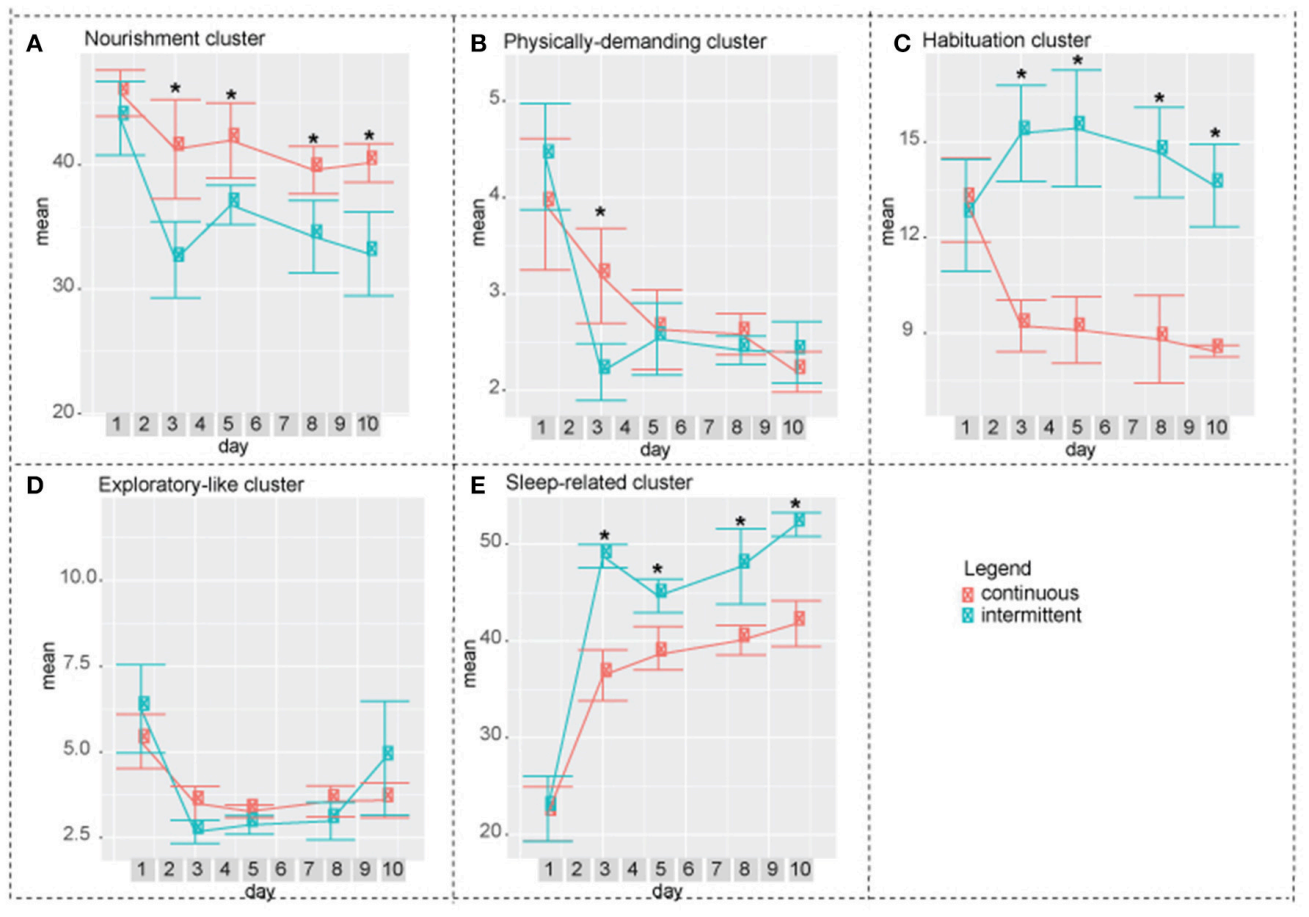
Difference between intermittent and continuously recorded mice by cluster**. (A)** Nourishment cluster. On days 3, 5, 8, and 10 continuously isolated mice spent more time performing activities in this cluster compared to intermittently isolated mire. **(B)** Physically demanding cluster. On day 3 continuously isolated mice spent significantly higher percentag of time performing activities in this cluster compared to the intermittenly isolated mice. **(C)** Habituation cluster. On days 3, 5, 8, and 10 intermittently isolated mice performed activities in this cluster significantly more compared to the continuously isolated mice. **(D)** Exploratory-like cluster. There was no significant difference between two groups. **(E)** Sleep-related cluster. Intermittently isolated mice spent significantly higher percentage of time performing activities in this cluster. Two-way ANOVA followed by Tukey HSD was used. *n* = 8.

**Figure 6:**
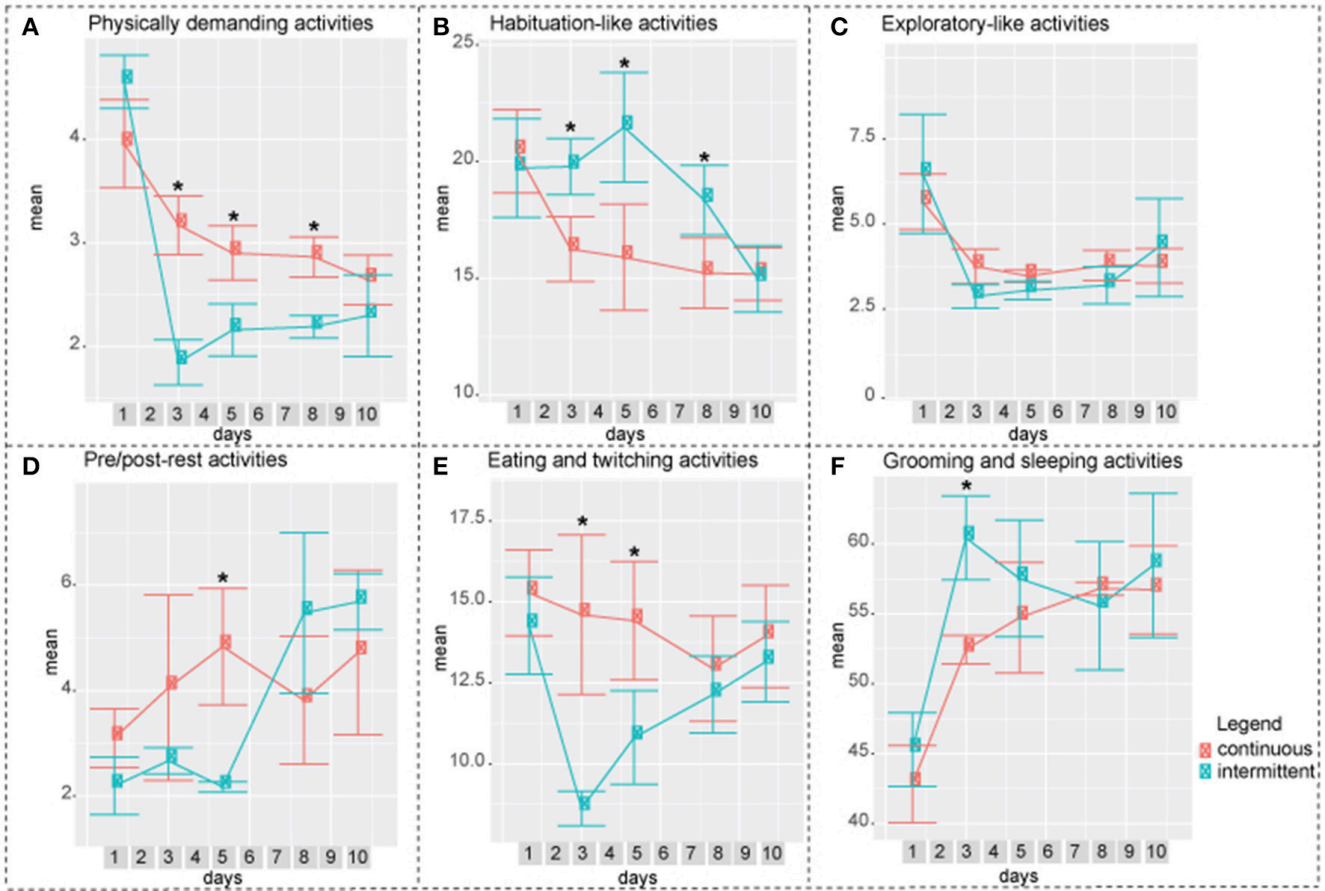
Difference between intermittently and continuously recorded mice by factor**. (A)** Factor 1: Physically demanding activities. Continuously recorded mice spent higher percentage of time performing these activities compared to intermittently recorded mice on days 3, 5, and 8. **(B)** Factor 2: Habituation-like activities. Intermittently recorded mice spent higher percentage of time performing these activities compared to continuously recorded mice on days 3,5, and 8. **(C)** Factor 3: Exploratory-like activities. There was no significant difference between the two groups. **(D)** Factor 4: Pre/post-rest activities. On day 5, continously recorded mice spend significantly higher percentage of time performing these activities. **(E)** Factor 5: Eating and Twitching. On days 3 and 5 continuously recorded mice spent significantly higher percentage of time on these activities. **(F)** Factor 6: Grooming and Sleeping. On day 3 intermittently isolated mice spent higher percentage of 24 h performing these activities. Two-way ANOVA followed by Tukey HSD was used. *n* = 8.

Here, we compare the behavioral array of two distinct groups: 1. Mice that were isolated into a home cage for a period of 11 days without any human intervention for this period. 2. Mice that were placed into isolation on day 1, 3, 5, 8, and 10 and were placed into groups of four for days 2, 4, 6, 7, and 9. All manipulations were done by a familiar female technician, who has been handling these mice since the arrival at the facility. Transitions between cages took no more then 30 s per animal. From literature, such careful animal manipulation does not statistically change behavioral output of mice. We did not observe any statistical changes associated with handling. As such, the variation in the behavioral array is attributed to the effect of intermittent socialization and re-introduction to a familiar home cage. Behavioral activities for intermittent and continuous recording paradigms separated by clusters are presented in Figure 5 and factors are presented in Figure 6.

In both, exploratory-like cluster and factor 3: exploratory-like activities, there were no significant differences between the two groups (Figures 5D,6C). Activities in the habituation-like cluster were performed significantly more by the intermittently isolated mice compared to the continuously isolated group (Figure 5C). Factor 2: habituation-like activities were similarly performed more by the intermittently isolated mice on days 3, 5, and 8 compared to the continuously isolated group (Figure 6B). Sleep-related cluster was significantly higher in the intermittently isolated group on days 3, 5, 8, and 10 compared to the continuously isolated group (Figure 5E). Factor 6: sleeping and grooming activities was significantly higher in intermittently isolated group on day 3 only (Figure 6F). Nourishment cluster was significantly higher in the continuously isolated group on days 3, 5, 8, and 10 compared to the intermittently isolated group (Figure 5A). Factor 5: eating and twitching activities were higher in the continuously isolated mice compared to the intermittently isolated mice on days 3 and 5 (Figure 6E). Activities in the physically-demanding cluster were exhibited for longer percentage of the 24 h period by the continuously isolated mice on day 3 only (Figure 5B). Factor 1: physically-demanding activities were exhibited more by continuously isolated mice compared to intermittent group on days 3, 5, and 8 (Figure 6A). Lastly, factor 4: pre/post-rest activities were performed significantly more by continuously isolated mice on day 5 only (Figure 6D).

## Discussion

Using automated supervised behavioral assessment software, we analyzed 24 h non-interrupted recordings of mice recorded continuously for a duration of 11 days and mice recorded intermittently, while being placed in groups of four vs. socially isolated. We used correlation matrix-based hierarchical clustering and factor analysis to separate the 33 activities into meaningful clusters and groups without losing the exhaustive nature of the datasets. Using this statistical approach, we were able to define several groups of behavioral activities that were significantly different between two experimental paradigms: continuously and intermittently recorded mice. We were able to clearly define acclimatization period in the home cage as 1 day. During this period variability of all activities was the highest.

### Acclimatization period

When an animal is placed in a new environment such as a new animal facility, a new home cage, an open field, a maze, or a rotarod, it undergoes a period of acclimatization. It is a common practice to allow a mouse to habituate itself to a novel environment before conducting any behavioral testing to ensure that the results obtained in the experiment will hold true throughout other environmental conditions. Each behavioral test has an acclimation period that was experimentally defined. For example, it is accepted to acclimate mice 1 h before a rotarod test (Kalueff et al., 2008), 5–15 min for a treadmill gait test (Hampton et al., 2004; Kale et al., 2004), 6 trails in 1 day for a water maze test (Vorhees and Williams, 2014), and 10 min/day for 3 consecutive days for social interaction testing (Barkus et al., 2012). Spruijt et al. monitored the percentage of time that C57Bl/6 mice spent moving during a 12 h period for the duration of 6 days. On the first day, mice moved roughly 30% of the time, whereas on days 2, 3, 4, 5, and 6 the level of activity ranged from 14 to 16% (Spruijt et al., 2014). Similar to his work, we found that day 1 was statistically different in all clusters and in factors 1, 2, 3, and 6, and as such, is the acclimatization period for home cage long-term behavioral assessments (Figures 3, 4). Furthermore, after 1 day of acclimatization the intermittently recorded mice do not exhibit the same acclimatization-like behavioral arrays during the following re-introduction days, while being put back and forth between socialization and isolation environments (Figures 5, 6).

### Social isolation

The majority of automated home cage video assessment software are designed to analyze a single animal long-term. Social isolation of mice was shown to inflict brain molecular changes and behavioral pattern changes (Matsumoto et al., 1991; Pibiri et al., 2008; Koike et al., 2009; Berry et al., 2012; Ieraci et al., 2016). Post-weaning (at 4 weeks old) social isolation was reported to cause the most severe and long-lasting changes, such as aggression, cognitive rigidity, hyper-locomotor activity, impaired fear memory, reduced prefrontal cortical volume, decreased cortical and hippocampal synaptic plasticity (Fone and Porkess, 2008; Ouchi et al., 2013). Ouchi et al. reported that 1 week of isolation causes irreversible spatial attention deficit in 4 weeks old mice (Ouchi et al., 2013). In adult mice, Ieraci et al. reported that after 31 days of solitary confinement mice exhibit anxiety and depression-like behaviors in open field test and tail suspension tests (Ieraci et al., 2016). The same group demonstrated that there is a correlation between these behavioral changes and the reduction of several neuroplasticity-related genes in hippocampus and prefrontal cortex. The brain-derived neurotrophic factor (BDNF) level, which is known to regulate behavioral shifts induced by stress, was found to be down regulated when the mouse undergoes 31 days isolation. Another group working with adult (3 months old) mice, demonstrated reduced levels of BDNF in the brain, increased levels of corticosterone, and increased anxiety and depressive-like behavioral after 4 weeks of isolation (Berry et al., 2012).

In our work, we demonstrated the gradual and mild effect of isolation that is detectible by 11 days post isolation in four clusters and three factors. Performance of physically demanding cluster gradually decreases reaching a statistically significant drop at day 7 (Figure 3B). In factor 1: physically demanding activities a similar trend is present, which becomes statistically significant at day 10 (Figure 4A). Activities in the exploratory-like cluster statistically increase at day 11 (Figure 3D), similar to factor 3: exploratory-like activities, which also sees an increase on day 11 (Figure 4C). Our work suggests a link between isolation and an increase in exploratory-like activities, as such, further research is needed to understand this phenomenon. Increased percentage of 24 h period was dedicated to sleep-related cluster, reaching a significant increase at day 11 (Figure 3E). Similarly, factor 6: grooming and sleeping activities were significantly increased by day 8 (Figure 4F). Lastly, factor 2: habituation-like activities did not reflect any statistical change over the 11 days of isolation (Figure 4B), yet habituation-like cluster decreased significantly by day 6 (Figure 3C). In part, we attribute this decrease to the decreased interest in the surrounding environment, which was rearranged and the nesting areas set up. We attribute this decrease in habituation-like activities to the effect of social isolation because as the intermittently recorded mice were re-introduced to their home cages, habituation-like activities in both clusters and factors showed significant increases.

### Socialization, re-introduction to the home cage, and handling

Human handling was shown to influence experimental results (Crabbe et al., 1999; van Driel and Talling, 2005; Gaskill et al., 2013; Sorge et al., 2014). The cocktail of chemicals within the body secretions of males (humans and other intact mammals) was shown to significantly effect the behavioral and molecular results of experiments, resulting in pain inhibition in mice and rats due to the increased plasma corticosterone levels (Sorge et al., 2014). It was shown that rats produce consistent results in various anxiety tests when the testing is conducted by a familiar experimenter. All other factors staying the same, unfamiliar experimenters produced inconsistencies between trials (van Driel and Talling, 2005). In our work, all manipulations were done by a familiar female technician for a length of 30 s on average per move per animal. We did not observe any consistent behavioral changes associated with handling in the intermittently isolated group.

The increased sleep-related cluster, which consists of sleeping, twitching, awaking, and pausing, can be explained by the effect of intermittent socialization the previous day. These results are in agreement with work by Lone et al. that reported increase in sleep activities due to short-term neuroplasticity following socialization in fruit flies (Lone et al., 2016). In response to the increased amount of time spent on sleeping activities, there was a decrease in physically demanding and nourishment clusters.

Re-introduction to a home cage was accompanied by a significant increase in the habituation cluster activities compared to the baseline of the continuously isolated mice. We attribute this increase to the need to set up the nesting space after being absent.

Looking at the overall factor analysis of the two paradigms: continuous and intermittent isolation, factors 1, 2, 4, 5, and 6 suggest that mice in the intermittent group adopted to the testing paradigm by day 10; at which time, there were no significant differences between the groups. On the other hand, the overall cluster analysis describes the data at a different angle. Nourishment, habituation, and sleep-related clusters remain statistically different between the two groups. As such, we conclude that there are some strong indications (factor analysis) that mice are adopting to the experimental paradigm by day 10, yet there are still observable variations between the groups. Even though the groupings by cluster and factor seem to be very similar, non-the-less both approaches present the data from different angles. As such, several statistical approaches are necessary to accurately interpret the multi-dimensional behavioral outputs. Exploratory-like cluster and the correlating factor 3: exploratory-like activities were the only behavioral outputs that showed no difference between the two paradigms. There was an acclimation effect on the first day, after which the trend remained constant. Considering the consistency of these activities throughout the experiment, the name “exploratory activities” might be misleading. Mice did not explore their cage environment each day, instead it would be more appropriate to think of it as surveillance activities. It appears that each day mice spent a predetermined amount of time sniffing, chewing, remaining reared up, coming down from partially reared, rearing up partially and coming down to partially reared or repetitively jumping. As such, this cluster and factor would be more appropriately described as regular surveillance check.

## Conclusion

In conclusion, this work interprets mouse home cage activities throughout a 24 h period and proposes a base line of a day-appropriate daily routine of a healthy C57Bl/6J mouse that can be used for various experimental paradigms, including disease, neuroinflammation, or drug testing to trace behavioral changes that follow intervention. We described the in-depth analysis of a long-term recording of mouse behavior, while maintaining the exhaustive nature of the dataset. Using an automated behavior recognition technology together with exploratory analysis performed in R programming, we were able to detect, with precision and minimal handling, the behavioral shifts that are associated with social isolation during an 11 days period; to define an acclimatization period required for 24 h home cage recording assessments; and to summarize the behavioral changes associated with intermittent socialization and re-introduction to a familiar home cage. Here, we propose a stream-line approach to the analysis of home cage behavior, we provide detailed description of codes adopted to R program environment that is aimed to help researchers to analyze behavioral data without adding exceeding costs to already costly animal experimentation (**SI 4**).

## Ethics statement

All experiments and procedures were approved by the University of Sherbrooke animal care and use committee.

## Author contributions

KY, DG, PG, and KG conceptualized the work. KY, DG, and KG formatted the figures, and wrote the manuscript. JS, PG, and DG conducted all data analysis in R. KY and KG collected and analyzed behavioral data prior to R. MG, SM, CS, and DH-M handled and recorded mice. TC added the labels on the video files, and contributed to the analysis. PG conducted a scientific proof reading of the manuscript.

### Conflict of interest statement

The authors declare that the research was conducted in the absence of any commercial or financial relationships that could be construed as a potential conflict of interest. The reviewer PSC and the handling editor declared their shared affiliation at the time of review.
