# Supplementary material for: Exhaustive Multi-Parametric Assessment of the Behavioral Array of Daily Activities of Mice Using Cluster and Factor Analysis
Source: Front Behav Neurosci. 2018 Aug 30;12:187. doi: 10.3389/fnbeh.2018.00187 (PMC6125369; doi:10.3389/fnbeh.2018.00187)
Supplement: Supplementary file 6 [file Presentation_1.PDF]

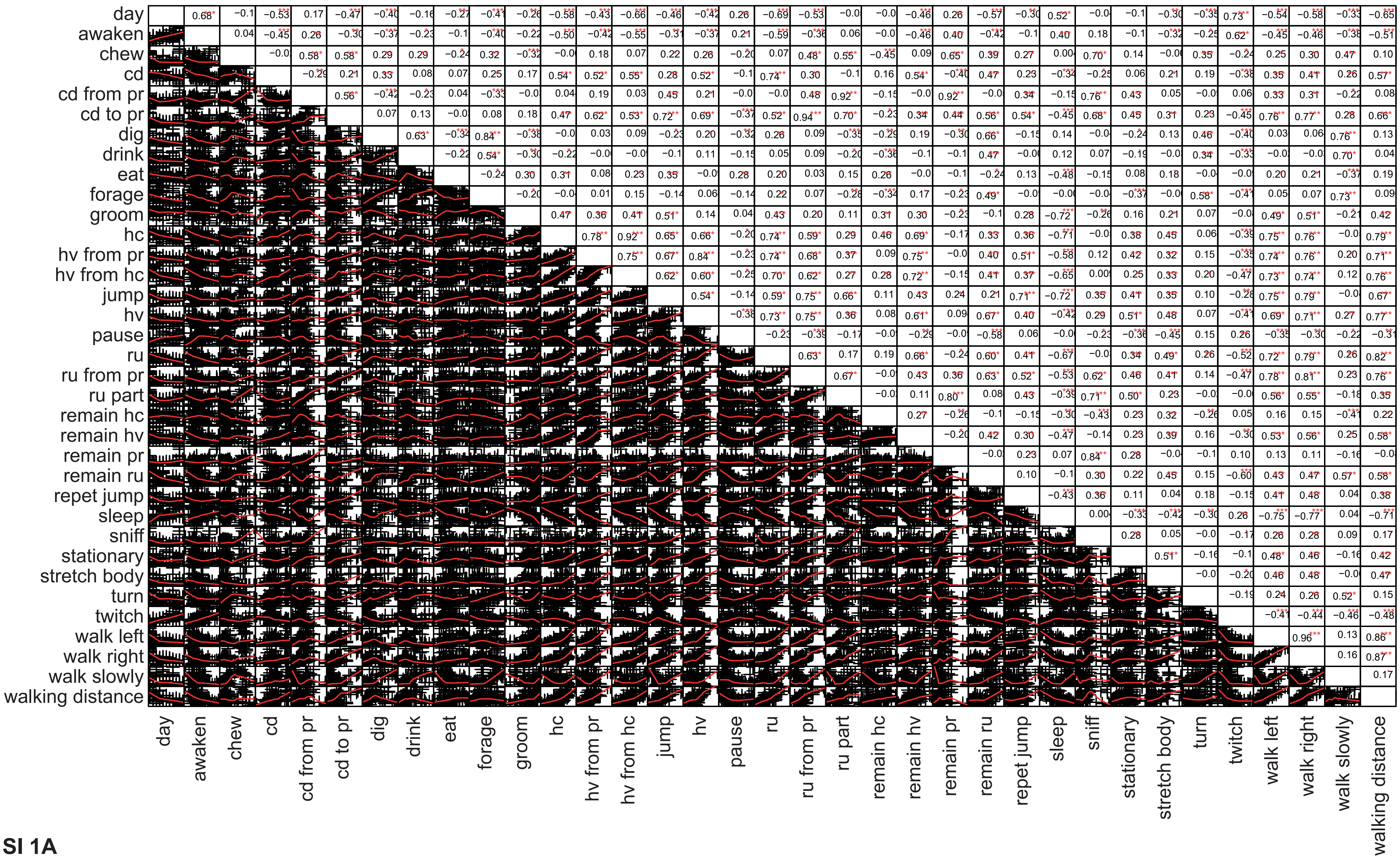
day
awaken
chew
cd
cd from pr
cd to pr
dig
drink
eat
forage
groom
hc
hv from pr
hv from hc
jump
hv
pause
ru
ru from pr
ru part
remain hc
remain hv
remain pr
remain ru
repet jump
sleep
sniff
stationary
stretch body
turn
twitch
walk left
walk right
walk slowly
walking distance

SI 1A

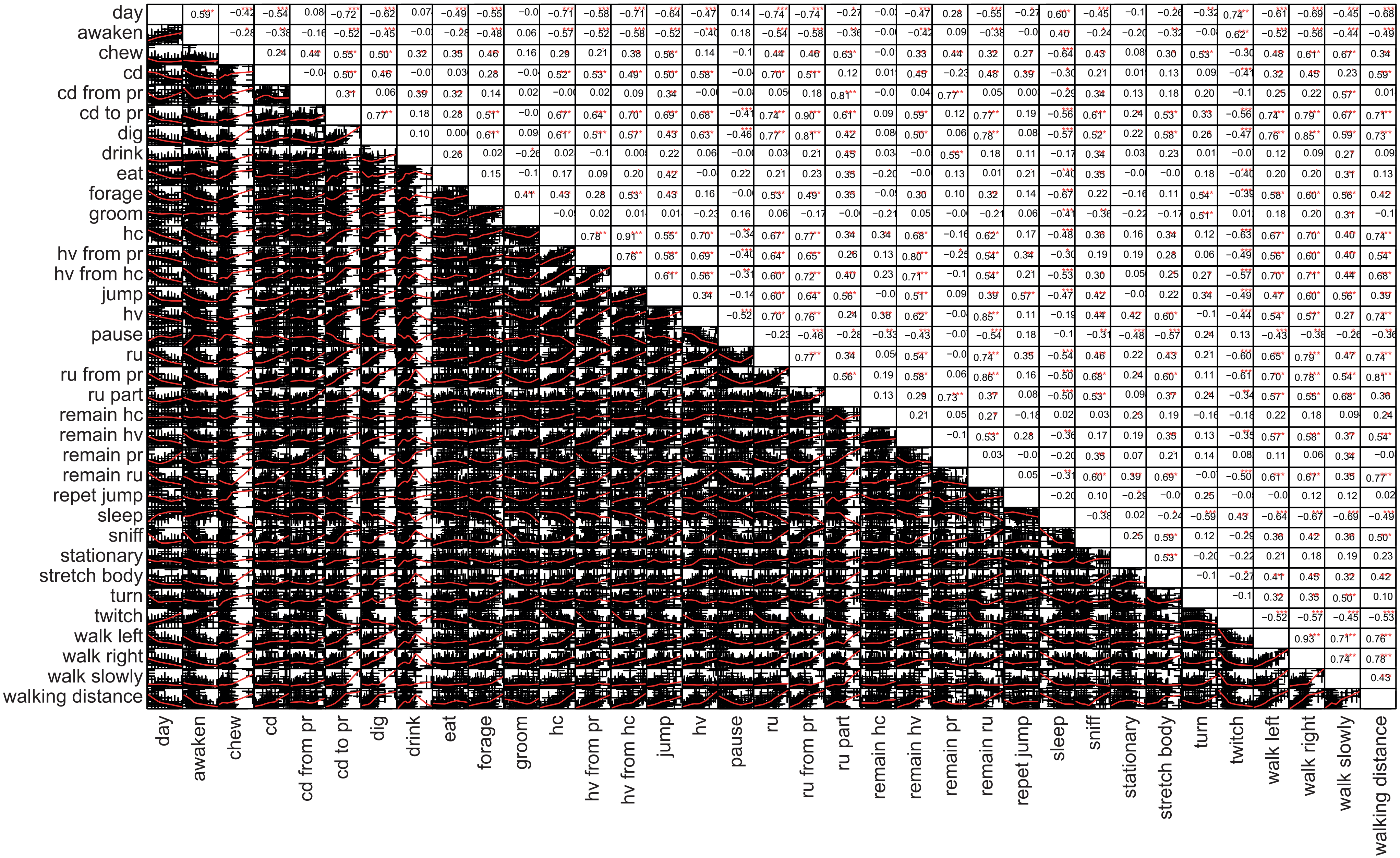
day
awaken
chew
cd
cd from pr
cd to pr
dig
drink
eat
forage
groom
hc
hv from pr
hv from hc
jump
hv
pause
ru
ru from pr
ru part
remain hc
remain hv
remain pr
remain ru
repet jump
sleep
sniff
stationary
stretch body
turn
twitch
walk left
walk right
walk slowly
walking distance

SI 1B

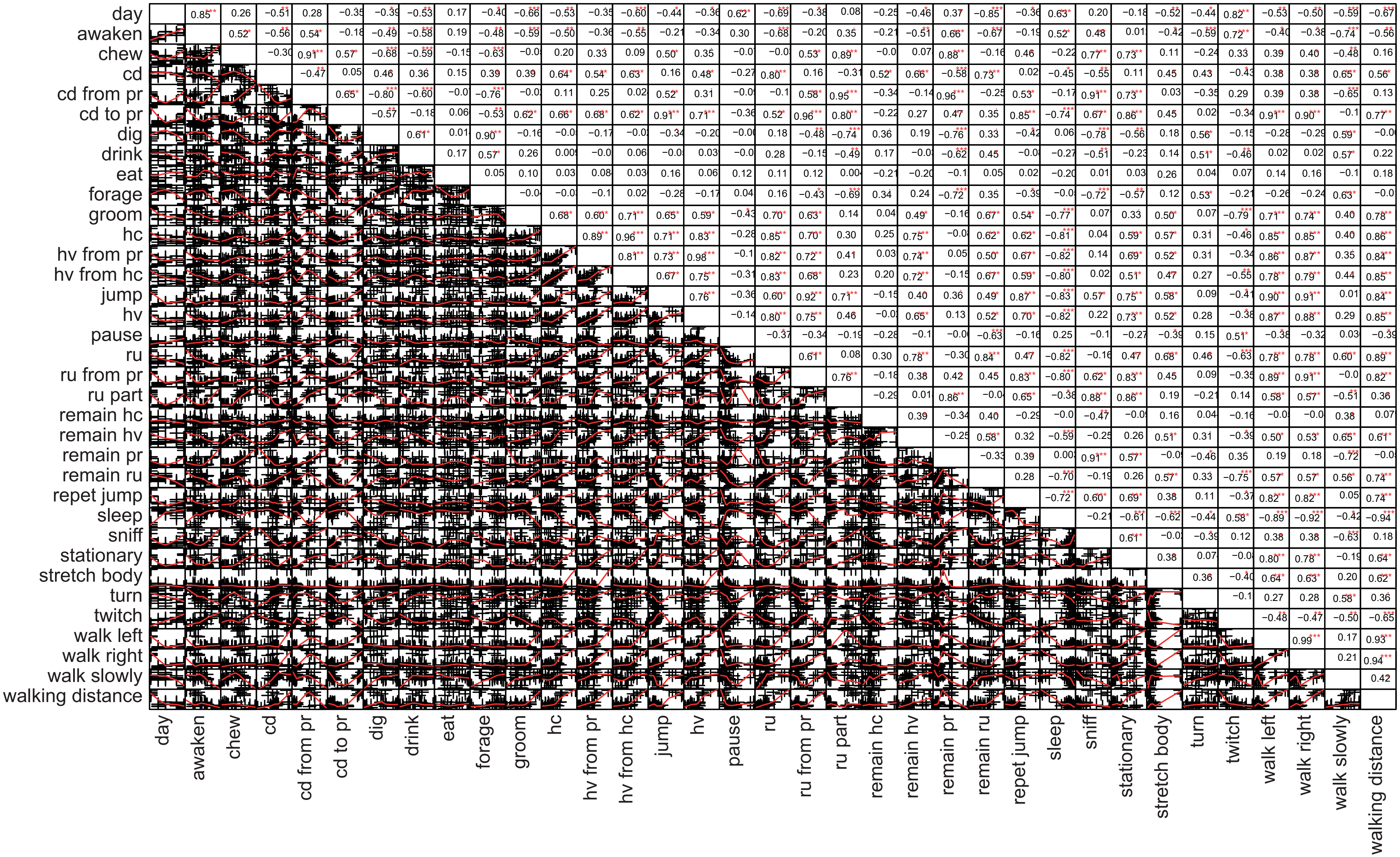

SI 1C.

SI 1. Correlation Matrix of Behavioral Activities of Mice. A) Combined dataset, B) Continuous dataset, C) Intermittent dataset. Correlation between time (24 hour segments), individual activities (expressed as percentage of 24 hours), and daily walking distance (in meters). Bivariate scatter plots with a fitted line are presented on the plot in the bottom of the diagonal, with the correlation value and significance (represented by red stars) in the top of the diagonal. *** is p ≤ 0.001, ** is p ≤ 0.01, * is p ≤ 0.05. n=8.

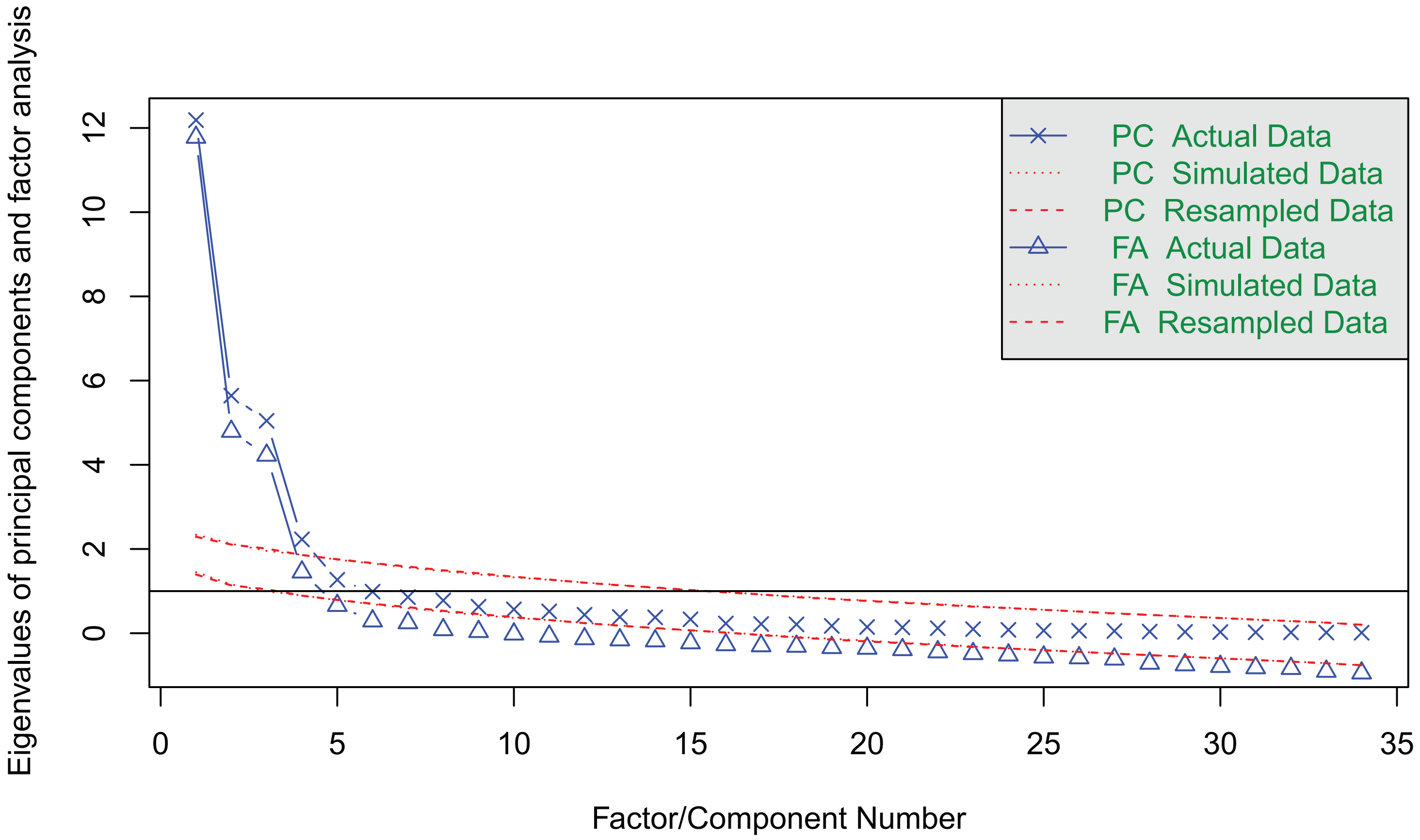

SI 2. Parallel Analysis Scree Plot. There are 6 suggested components and 6 factors according to the parallel analysis. Here, the eigenvalues of the correlation matrix of the complete dataset were compared with a matrix of randomly generated values of the same dimensionality.

Loadings:

	MR1	MR3	MR2	MR6	MR4	MR5
awaken	-0.492					
chew		0.751	0.469			
cd	0.674					
cd_from_pr		0.925				
cd_to_pr	0.523	0.666				
dig			0.883			
drink1			0.706			
eat1				0.415		0.426
forage			0.880			
groom				0.633		
hang_cuddled	0.874					
hang_vert_from_ru	0.896					
hang_vert_from_hang_cuddled	0.856					
jump	0.624	0.426		0.438		
land_vert	0.774					
pause					-0.653	
rear_up	0.808					
ru_full_from_partial	0.622	0.574				
ru_partially		0.877				
remain_hang_cuddled			-0.470			
remain_hang_vert	0.803					
remain_part_reared		0.934				
remain_ru	0.452		0.561		0.538	
repet_jumping	0.486					
slp	-0.558			-0.766		
sniff		0.902				
stationary					0.533	
stretch_body					0.640	
turn			0.619			
twitch	-0.415		-0.430			-0.451
walk_left	0.667			0.479		
walk_right	0.712			0.483		
walk_slowly			0.877			
walk_dist	0.760					

	MR1	MR3	MR2	MR6	MR4	MR5
SS loadings	9.088	5.860	5.081	2.779	2.620	1.012
Proportion Var	0.263	0.169	0.146	0.086	0.081	0.034
Cumulative Var	0.263	0.432	0.578	0.664	0.745	0.779

SI 3. Print Screen from R of the Complete Dataset Factor Loadings. Resulting table of factor analysis with a cutoff of λ=0.4. Most factors load uniquly to a single activity, which explains the lack of correlation between factors. Twitch, remain reared up, and jump have three factors with loadings over 0.4, which denotes that these activities explain partially the variance in each of the factors.

SI 4

These are simplified codes for beginners in R programing accessible to anyone with minimal experience in R. General understanding of data arrangements and naming variable in R is necessary. The codes may seem redundant and can be improved by writing function modules. All commands and outputs are given for example purposes only. In grey are the subheadings, in green are the outputs from the program, # separates codes from explanations, in turquoise are the corresponding figures generated using the code.

Download R and R studio:

Install the following packages using install.packages() command:

```
install.packages("xlsx")  # to read an Excel file
install.packages("ggplot2") # to build figures
install.packages("dplyr") # to select parts of the data
install.packages("reshape") # to restructure the data
install.packages("PerformanceAnalytics") # to build correlation matrices
install.packages("corrplot") # to analyze correlation
install.packages("psych") # to run factor analysis
install.packages("GPArotation") # to run parallel analysis
install.packages("nFactors") # to validate factor analysis
install.packages("devtools") # to close pdf rendering of graphs
install.packages("rJava") # to load files using interactive window
```

Load all installed packages using library() command:

```
library ("xlsx")  # to read an Excel file
library ("rJava") # to load files using interactive window
library ("ggplot2") # to build figures
library ("dplyr") # to select parts of the data
library ("reshape") # to restructure the data
library (“PerformanceAnalytics") # to build correlation matrices
library ("corrplot") # to analyze correlations
library ("psych") # to run factor analysis
library ("GPArotation") # to run parallel analysis
library ("nFactors") # to validate factor analysis
library ("devtools") # to close pdf rendering of graphs
```

Loading the data from excel file sheet 1

```
df <- read.xlsx (choose.files(), sheetIndex = 1, header = TRUE)
# in the open window, browse and choose a file
head(df) # to verify the data, this command shows the first 6 rows of data
```

```
  id type day     pause stretch_body  stationary
1  2   in   1 0.9723729    1.4139932 0.059631213
2  2   in   3 2.0458917    0.1772661 0.003594702
3  2   in   5 1.8821950    0.2890682 0.010493969
4  2   in   8 2.5498240    0.1080865 0.010907048
5  2   in  10 3.7784508     0.1059594 0.069309882
```

```
6  3   in   1 1.1571854    1.4490153 0.094354112
```

The definition of the experimental group is in the first three columns, followed by the variables (pause, stretch, stationary).

srt(df) # to look at the structure of the data

```
data.frame':           63 obs. of  7 variables:
 $ id          : num  2 2 2 2 2 3 3 3 3 3 ...
 $ type        : chr  "in" "in" "in" "in" ...
 $ day         : num  1 3 5 8 10 1 3 5 8 10 ...
 $ pause       : num  0.972 2.046 1.882 2.55 3.778 ...
 $ stretch_body: num  1.414 0.177 0.289 0.108 0.106 ...
 $ stationary  : num  0.05963 0.00359 0.01049 0.01091 0.06931 ...
```

To select only the data that has variability (some activities may have constant values like “0” or “1”), use the following set of commands:

```
info<-wth_c_i[,1:3] # to select a description of each observation
numbers<-wth_c_i[,4:7] # to select variables
```

To take out zero variance

```
nzv<-nearZeroVar(numbers, saveMetrics = TRUE)
print(paste('Range:', range(nzv$percentUnique)))
Zero<-nzv[nzv$percentUnique > 3, ]
df_w<- df[c(rownames(Zero))]
working<-cbind(info, df_w) # this data frame has all activities with zero variance removed
```

To select data based on the “type”, continuous vs intermittent recordings

```
cont<-working[working$type %in% "con", ] # to select the data from continuous recordings
int<-working[working$type %in% "in", ] # to select the data from intermittent recordings
```

Correlation analysis

```
cont_d<-scale(cont[,4:7]) # to select variables only and to scale the data
corr_comb_cont<-cor(as.matrix(cont_d, "pearson")) # to run correlation analysis
c_corr_comb_cut<-which(abs(corr_comb_cont) > 0.45, arr.ind=TRUE) # to remove all r^2 > 0.45
str(c_corr_comb_cut) # to extract the number of significant correlations in the data frame
```

Plot correlation matrix (SI 1)

```
pdf("con_d.pdf", width = 8, height = 5, pointsize = 12) # to start writing a pdf file
chart.Correlation(con_d, method = "pearson",  histogram =FALSE , pch=3) # to build the matrix
dev.off() # to close the pdf
```

Factor analysis for the scaled variables of the whole data set
Before running factor analysis, use parallel analysis to choose the number of factors (SI 2)

```
fa.parallel(raw_casted_working) # to determine the appropriate number of factors and principle components
```

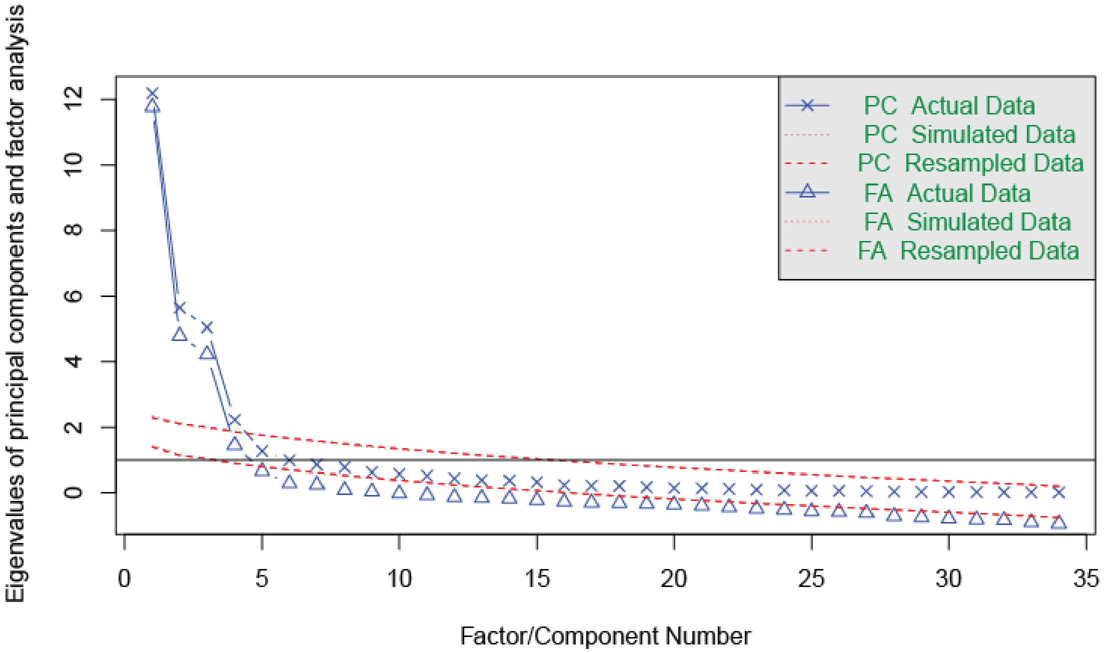

vss(days_only, n=4, rotate = "oblimin", fm = "pa") # statistics across the possible number of factors

```
Very Simple Structure
Call: vss(x = s_combic, n = 9, rotate = "varimax", fm = "pa")
VSS complexity 1 achieves a maximimum of 0.69  with  3  factors
VSS complexity 2 achieves a maximimum of 0.85  with  5  factors

The Velicer MAP achieves a minimum of 0.06  with  9  factors
BIC achieves a minimum of  -70.33  with  7  factors
Sample Size adjusted BIC achieves a minimum of  895.29  with  9  factors

Statistics by number of factors
  vss1 vss2   map dof chisq     prob sqresid  fit RMSEA    BIC SABIC complex eChisq
SRMR eCRMS  eBIC
1 0.60 0.00 0.076 527  3997  0.0e+00    63.2 0.60  0.28 1565.0  3229     1.0   4315
0.195 0.201  1883
2 0.67 0.76 0.070 494  3340  0.0e+00    37.5 0.76  0.26 1059.8  2620     1.3   2290
0.142 0.151    10
3 0.69 0.81 0.067 462  3026  0.0e+00    25.1 0.84  0.26  894.2  2353     1.4   1441
0.113 0.124  -692
4 0.65 0.85 0.067 431  2656 5.0e-316    16.6 0.89  0.25  667.2  2028     1.6    866
0.087 0.100 -1123
5 0.59 0.85 0.071 401  2251 1.9e-254    11.4 0.93  0.24  400.4  1667     1.7    518
0.068 0.080 -1333
6 0.50 0.82 0.077 372  1981 3.4e-217     8.5 0.95  0.23  264.2  1439     1.9    336
0.054 0.067 -1381
7 0.53 0.80 0.058 344  1517 1.1e-146     6.0 0.96  0.21  -70.3  1016     1.9    185
0.040 0.052 -1403
8 0.52 0.79 0.060 317  1459 1.2e-145     4.1 0.97  0.21   -4.1   997     1.9    115
0.032 0.042 -1348
9 0.53 0.77 0.056 291  1319 1.6e-130     3.2 0.98  0.21  -23.8   895     2.0     69
0.025 0.034 -1274
```

fa_results <- fa(r = s_com_d,
  fm = "minres",
  nfactors=6,

```
        rotate = "varimax")
fa_results
```

Factor Analysis using method = minres

Call: fa(r = s_combic_d, nfactors = 7, rotate = "varimax", fm = "minres")

Standardized loadings (pattern matrix) based upon correlation matrix

	MR1	MR3	MR2	MR6	MR4	MR5	MR7	h2	u2	com
awaken	-0.49	0.21	-0.35	-0.18	-0.17	-0.32	0.02	0.57	0.425	3.8
chew	0.08	0.75	0.47	-0.11	-0.01	-0.11	-0.07	0.82	0.181	1.8
cd	0.67	-0.27	0.20	-0.02	0.03	0.09	0.05	0.58	0.421	1.6
cd_from_pr	0.00	0.93	-0.26	0.07	0.04	-0.06	-0.05	0.94	0.062	1.2
cd_to_pr	0.52	0.67	0.21	0.18	0.23	0.06	0.26	0.92	0.082	3.0
dig	0.10	-0.19	0.88	-0.20	0.20	0.02	-0.08	0.91	0.089	1.4
drink1	-0.09	-0.03	0.71	-0.07	0.08	0.07	0.23	0.58	0.425	1.3
eat1	0.11	-0.06	-0.32	0.41	-0.12	0.43	-0.06	0.49	0.513	3.3
forage	0.09	-0.11	0.88	-0.02	-0.08	0.09	-0.19	0.84	0.155	1.2
groom	0.36	-0.16	-0.28	0.63	-0.01	-0.02	0.06	0.64	0.364	2.2
hang_cuddled	0.87	-0.01	-0.17	0.22	0.16	0.20	-0.19	0.94	0.057	1.5
hang_vert_from_ru	0.90	0.17	0.00	0.11	0.08	-0.15	0.08	0.88	0.120	1.2
hang_vert_from_hang_cuddled	0.86	0.05	0.04	0.18	0.07	0.23	-0.22	0.87	0.130	1.4
jump	0.62	0.43	-0.16	0.44	0.03	0.15	0.18	0.84	0.156	3.2
land_vert	0.77	0.22	0.11	-0.05	0.39	-0.12	0.21	0.87	0.132	2.0
pause	-0.20	-0.13	-0.17	0.18	-0.65	0.03	0.04	0.55	0.450	1.6
rear_up	0.81	-0.07	0.19	0.28	0.23	0.12	0.11	0.85	0.151	1.7
ru_full_from_partial	0.62	0.57	0.15	0.15	0.30	0.16	0.22	0.92	0.076	3.2
ru_partially	0.21	0.88	-0.23	0.22	0.14	0.04	-0.07	0.94	0.062	1.5
remain_hang_cuddled	0.22	-0.33	-0.47	0.14	0.28	0.08	-0.33	0.59	0.412	4.3
remain_hang_vert	0.80	-0.08	0.08	0.02	0.14	-0.17	-0.21	0.75	0.250	1.3
remain_part_reared	-0.17	0.93	-0.15	-0.11	0.03	-0.08	-0.05	0.95	0.051	1.2
remain_ru	0.45	0.11	0.56	-0.21	0.54	0.14	0.15	0.91	0.093	3.6
repet_jumping	0.49	0.37	-0.04	0.20	-0.28	-0.03	0.33	0.61	0.394	3.9
slp	-0.56	-0.08	0.06	-0.77	-0.05	-0.13	0.06	0.93	0.070	2.0
sniff	-0.02	0.90	0.10	-0.13	0.12	0.09	0.23	0.91	0.085	1.3
stationary	0.28	0.31	-0.28	0.14	0.53	-0.07	0.03	0.56	0.438	3.1
stretch_body	0.31	0.01	-0.06	0.21	0.64	0.08	-0.05	0.56	0.438	1.8
turn	0.14	0.05	0.62	0.36	-0.27	-0.13	-0.12	0.64	0.358	2.4
twitch	-0.42	-0.06	-0.43	-0.01	-0.17	-0.45	-0.07	0.60	0.401	3.4
walk_left	0.67	0.33	0.07	0.48	0.32	0.05	-0.01	0.89	0.109	2.9
walk_right	0.71	0.32	0.09	0.48	0.28	0.06	0.02	0.94	0.064	2.7
walk_slowly	0.18	0.00	0.88	-0.10	0.01	-0.11	0.08	0.83	0.170	1.2
walk_dist	0.76	0.11	0.09	0.31	0.33	0.17	0.12	0.85	0.153	2.0

	MR1	MR3	MR2	MR6	MR4	MR5	MR7
SS loadings	8.96	5.73	4.95	2.65	2.49	0.88	0.80
Proportion Var	0.26	0.17	0.15	0.08	0.07	0.03	0.02
Cumulative Var	0.26	0.43	0.58	0.66	0.73	0.75	0.78
Proportion Explained	0.34	0.22	0.19	0.10	0.09	0.03	0.03

```
Cumulative Proportion 0.34 0.56 0.74 0.84 0.94 0.97 1.00
```

```
print(fa_results$loadings, cut=0.4) # to show only loading values of lambda 0.4 cut off (SI 3)
pdf("fa_s_com_d.pdf") # to build a figure of factors open pdf
diagram(fit = fa_results,
        main = "Factor Analysis")
dev.off() # to close the pdf
```

To select data according to the list of activities generated in factor analysis and to make bar graphs (shown below is an example of the code for factor 1) Figure 3 and 4

```
factor_1<-select(working, c(id, type, day,  hang_vert_from_hang_cuddled, remain_hang_vert, hang_cuddled, walk_right, walk_left, rear_up, cd_from_pr, cd, land_vert, jump, ru_full_from_partial, awaken, repet_jumping))
factor_1_m<-melt(factor_1, id = c("id", "type", "day")) # to rearrange data for graphic
```

To generate bar graphs using only continuous dataset

```
plot_factor_1_bar<- ggplot(data=subset(factor_1_m[factor_1_m$type %in% "con", ]), aes(type, value))+
  geom_bar( stat = "summary", fun.y=mean, aes(fill=variable)) +
  facet_grid(~ day) + theme(legend.title=element_blank(),axis.title.x=element_blank())

pdf("factor_1_bar.pdf") # to open pdf
plot_factor_1_bar
dev.off() # to close pdf
```

Repeat the above steps for all factors

To build line graphs showing both intermittent and continues data Figure 5 and 6
Calculate mean and standard deviation for all behavioral activities within a factor

```
str(factor_1)
factor_1$total<-rowSums(factor_1[, 4:15])
factor_1_t<-select(factor_1, c(type, day, total))
factor_1_m<-melt(factor_1_t, id = c("type", "day"))
factor_1_m_sd<-summarise(group_by(factor_1_m, day,type),
                         mean=mean(value), sd=sd(value))
```

To build line graphs

```
plot_factor_1_line<- ggplot(data=factor_1_m_sd, aes(x= day, y=mean, color=type)) +
  geom_point(aes())+
  geom_line(aes()) +
  labs(title = "factor 6")+
  geom_errorbar(aes(ymin=mean-sd, ymax=mean+sd))+
  theme(legend.title=element_blank(),axis.title.x=element_blank())
pdf(plot_factor_1_line.pdf) # to open pdf
plot_factor_1_line
dev.off() # to close pdf
```

To build hierarchical clustering dendrogram and correlation matrix of the dataset Figure 1 and SI 1

```
corr_percentages <- cor(c_i_clust)
corrplot(corr = corr_percentages,
         method = "color",
         order = "hclust",
         type = "lower",
         col = colorRampPalette(c("blue","white","red"))(200),
         tl.col= "black",
         tl.cex = 0.5
)

distance_matrix <- dist(x = corr_percentages,
                        method = "euclidian")
clustering <- hclust(d = distance_matrix,
                     method = "complete")
dendrogram <- as.dendrogram(object = clustering)
rownames(corr_percentages[clustering$order,])
par(mar=c(0,0,0,20))
plot(dendrogram, horiz=TRUE)
order <- rev(clustering$order)
corr_percentages <- corr_percentages[order,]
corr_percentages <- corr_percentages[,order]
layout(matrix(c(1,1,2,2), nrow=2, ncol=2), widths=c(0.1,0.8))
```

To set graphical parameters of the dendrogram

```
par(cex=0.5, # cex is for the size of the text
    mar=c(3,0,12.5,0)) # mar is for the margins of the plot
```

To plot the dendrogram Figure 1

```
pdf("hclust_i_c1.pdf", width = 8, height = 5, pointsize = 12)
plot(dendrogram,
     axes=FALSE,  # Use axes = FALSE to remove the axes from the dendrogram
     horiz = TRUE, # horiz = TRUE to plot a horizontal dendrogram
     leaflab = "none") # leaflab = "none" to remove names from the tips of the dendrogram
dev.off() # to close the pdf file
```

ANOVA between days and types (intermittent vs continuous) of recordings to analyze the differences in factors

```
F_1<-subset(factor_1_t[factor_1_t$day%in% c(1, 3, 5, 8, 10), ] ) # to choose the data
F_1$day<-as.character(con_clust_1$day) # to convert days from numerical to character

model_s <- aov(formula=total~day*type, data = F_1)
summary(model_s)
```

To run posthoc test

```
TukeyHSD(model_s)
clust_p<-TukeyHSD(model_s)
res_c<-clust_p$`day:type`
res_c<-as.data.frame(res_c)
p_c_1<-subset(res_c[res_c$`p adj`< 0.05, ]) # to acquire a table of significant values only
```

p_c_1

	diff	lwr	upr	p adj
10:con-1:con	-1.3129862	-1.82943227	-0.7965401	1.167168e-09
3:con-1:con	-0.7848216	-1.26525102	-0.3043922	6.768606e-05
5:con-1:con	-1.0545225	-1.57096861	-0.5380764	5.098918e-07
8:con-1:con	-1.0917074	-1.60815351	-0.5752613	2.122613e-07
1:in-1:con	0.5988613	0.08241517	1.1153073	1.161889e-02
3:in-1:con	-2.1091011	-2.62554716	-1.5926550	0.000000e+00
5:in-1:con	-1.7967143	-2.31316040	-1.2802682	0.000000e+00
8:in-1:con	-1.7653174	-2.28176351	-1.2488713	0.000000e+00
3:con-10:con	0.5281646	0.02683726	1.0294919	3.132200e-02
1:in-10:con	1.9118474	1.37590600	2.4477889	0.000000e+00
10:in-10:con	0.9997800	0.46383857	1.5357214	4.316170e-06
3:in-10:con	-0.7961149	-1.33205632	-0.2601735	3.702288e-04
1:in-3:con	1.3836829	0.88235556	1.8850102	8.488943e-11

Video Clip Legends

Video Clip 1. Group of Physically Demanding Activities Exhibited in a Sequence by a Mouse. Mouse exhibits activities that are grouped in the physically demanding cluster and factor 1: physically demanding activities in a sequence. The following activities are played out in the video: rear up from partially reared, remain rear up, hang vertically from rear up, hand cuddled, hang vertically from hang cuddled, walk right, and walk left. Video speed is 12 frames per second.

Video Clip 2. Group of Exploratory-like Activities Exhibited in a Sequence by a Mouse. Mouse exhibits activities that are grouped in the exploratory-like cluster and in factor 3: exploratory-like activities in a sequence. The following activities are play out in the video: come down from partially reared, come down to partially reared, rear up partially, remain partially reared, rear up from partially reared, and sniff. There are two fragments put together in the video clip because the sequences of exploratory-like activities are very short in length (about 5 seconds each). Video speed is 12 frames per second.

Video Clip 3. Group of Habituation-like Activities Exhibited in a Sequence by a Mouse. Mouse exhibits activities that are grouped in the habituation-like cluster and in factor 2: habituation-like activities. The following activities are played out in the video: forage, dig, and turn. Video speed is 12 frames per second.

Video Clip 4. Group of Nourishment Activities Exhibited in a Sequence by a Mouse. Mouse exhibits activities that are grouped in the nourishment cluster and in factor 5: eat and twitch. The following activities are played out in the video: eat and chew. Video speed is 12 frames per second.

Video Clip 5. Group of Sleep-Related Activities Exhibited in a Sequence by a Mouse. Mouse exhibits activities that are grouped in the sleep-related cluster and the negatively correlated activities in factors 1, 4, 5, and 6; awaken, pause, twitch, and sleep respectively. The following activities are played out in the video: sleep, twitch, awaken, and pause. Video speed is 100 frames per second.
